# Arthroscopic-Assisted vs. Fluoroscopic-Only ORIF of Distal Radius Fractures: Clinical and Economic Perspectives

**DOI:** 10.3390/medicina61050796

**Published:** 2025-04-25

**Authors:** Wolfram Demmer, Antonina Jakob, Fabian Gilbert, Benedikt Fuchs, Sinan Mert, Nikolaus Wachtel, Riccardo Giunta, Verena Alt

**Affiliations:** 1 Department of Hand, Plastic and Aesthetic Surgery, Ludwig-Maximilians-University Munich, 80539 Munich, Germany; 2 Manager OR-Nursing Department, Ludwig-Maximilians-University Munich, 80539 Munich, Germany; 3 Department of Orthopaedics and Trauma Surgery, Musculoskeletal University Center Munich (MUM), University Hospital, Ludwig-Maximilians-University Munich, Ziemssenstr. 5, 80336 Munich, Germany; 4 Kreisklinikum Ebersberg, 85560 Ebersberg, Germany

**Keywords:** intra-articular distal radius fracture, arthroscopy, fluoroscopy, cost-effectiveness, ORIF, hand surgery, surgery of the wrist

## Abstract

*Background and Objectives*: Distal radius fractures (DRFs) are among the most common fractures globally, with a lifetime incidence of around 9%. They typically present in two age peaks: high-impact trauma in patients under 40 and low-energy trauma in those over 40. Intra-articular DRFs are classified according to the *Arbeitsgemeinschaft für Osteosynthesefragen* (AO) classification, influencing the treatment approach. Surgical management, particularly open reduction and internal fixation (ORIF) using volar plate osteosynthesis, is considered the gold standard. This study aims to compare the treatment costs of fluoroscopy-assisted ORIF and arthroscopy-assisted ORIF for intra-articular DRF. The analysis includes surgical procedure costs, material expenses, and operating time to evaluate the cost-effectiveness of both methods, considering reimbursement within the German healthcare system. *Materials and Methods*: A retrospective, monocentric study was conducted at Ludwig-Maximilians-University (LMU) Hospital, a supraregional hand trauma center in southern Germany. Patients with DRFs requiring ORIF were treated either with fluoroscopy or arthroscopic assistance. Group 1 included patients treated by the Department of Hand Surgery (Plastic Surgery), subdivided into Group 1a (arthroscopy-assisted) and Group 1b (fluoroscopy-only). Group 2 comprised patients treated by Orthopaedics and Trauma Surgery (fluoroscopy-only). Costs associated with surgical procedures, including materials, operating time, and postoperative care, were analyzed. *Results*: A total of 43 DRFs were treated. Group 1 consisted of 17 cases, with an average age of 49.6 years (SD = 19.4) and a 64% majority of female patients. Of these, 10 cases were treated with arthroscopy-assisted ORIF (Group 1a) and 7 with fluoroscopy-only ORIF (Group 1b). In Group 1a, the average age was 53.9 years (SD = 16.3) with 60% female and 40% male patients, while in Group 1b, the average age was 43.6 years (SD = 23.1) with 71.4% female patients. Group 2 included 25 cases, with an average age of 54.2 years (SD = 21.0) and a distribution of 64% female and 36% male patients. There was no significant difference in age and gender distribution within the groups and subgroups (*p* > 0.05). The mean procedure time was longer for arthroscopically assisted ORIF (111.5 min) compared to fluoroscopy-only ORIF (80.1 min), and even longer compared to Group 2 (65.0 min). Material costs were slightly higher in Group 1. Total costs for Group 1 averaged EUR 4906.58, with subgroup costs of EUR 5448.24 for arthroscopy-assisted and EUR 4132.80 for fluoroscopy-only. In comparison, Group 2 costs averaged EUR 3344.08. *Conclusions*: Intra-articular DRFs with severely displaced fragments or concomitant injuries benefit from arthroscopically assisted fracture treatment. While material costs do not significantly differ between arthroscopically assisted and fluoroscopy-only treatments, the significantly longer procedure time for arthroscopy-assisted ORIF results in the largest cost component. Despite this, reimbursement through the DRG system remains fixed and does not account for the increased operative duration or complexity of arthroscopic procedures. Our findings demonstrate that DRF treatment, regardless of the method used, is either not or only marginally cost-covering under the current German reimbursement structure. In the context of the ongoing shift towards outpatient hand surgery, including the management of DRF, adequate reimbursement rates are necessary to ensure the economic viability of DRF management, particularly for complex intra-articular fractures requiring arthroscopic assistance.

## 1. Introduction

Distal radius fractures (DRFs) are among the most common fractures in humans, with a lifetime incidence of approximately 9% [1]. Two age peaks can be identified, each differing in its trauma mechanism. In patients under 40 years of age, distal radius fractures are often caused by high-impact trauma. In contrast, in individuals over 40 years of age, low-energy trauma is the predominant cause of DRFs. Women in this group are significantly more affected, with a 6.2-fold higher risk [2,3]. Intra-articular DRFs are classified as 23-B1 to C3 fractures according to the *Arbeitsgemeinschaft für Osteosynthesefragen* (AO) classification [2,4]. The classification of the fracture, along with possible dislocation and associated injuries, determines the indication for surgical treatment. In recent years, there has been a clear trend toward operative management [5]. The gold standard for surgical treatment is open reduction and internal fixation (ORIF) with volar plate osteosynthesis, which achieves excellent outcomes [2,6,7]. In complex fractures with multiple or difficult-to-reduce fragments, additional procedures may need to be applied if necessary to achieve optimal postoperative outcomes. Also, ligamentous or chondral concomitant injuries can be addressed in a single-stage treatment [5,8,9,10]. In intra-articular fractures, especially in cases with radiologically detectable joint step-offs of >2 mm and suspected ligament injuries, ORIF combined with wrist arthroscopy is indicated [11,12,13].

The economic impact of distal radius fractures extends beyond direct medical costs [14,15]. These fractures can lead to significant indirect costs, including lost work hours and reduced independence, particularly in the elderly population. Additionally, they can result in long-term disability and a decreased quality of life [14,15,16]. Key cost drivers for surgical treatment of distal radius fractures include both surgical and patient factors, such as postoperative hospital admissions, simultaneous treatment of associated injuries, as well as increased operative time [17].

The reimbursement for hand surgery services for the treatment of distal radius fractures is difficult to compare due to differences in reimbursement within national healthcare systems. For example, the average cost for surgical treatment of DRFs in the USA in 2018 ranged between USD 6577 and USD 8181 when treated surgically [18]. In Germany, reimbursement depends on whether patients are treated inpatient or outpatient. Currently, slightly more than half of all DRFs are treated inpatient in Germany [19]. There is an increasing pressure to shift the treatment of distal radius fractures to an outpatient setting. The current Outpatient Surgery Contract of the National Association of Statutory Health Insurance Physicians of Germany does not include ORIF of distal radius fractures. Therefore, this procedure can generally only be performed under inpatient conditions [20]. However, based on personal experience, for otherwise healthy patients, cost coverage requests are usually submitted to and reviewed by the Medical Service of the Health Insurance Funds. In the context of this development, the necessary treatment must be evaluated from both medical and economic perspectives to justify additional treatment costs, such as those associated with concomitant wrist arthroscopy.

In Germany, the treatment of DRFs is traditionally carried out by orthopedic surgeons as well as plastic surgeons. Surgeons from both specialties can supplement their expertise with an additional qualification in hand surgery [21,22]. In the study setting, treatment of DRFs was provided by both the Department of Hand Surgery, Plastic Surgery, and Aesthetic Surgery, as well as the Department of Orthopaedics and Trauma Surgery. Both departments treated intra-articular DRFs, whereas only the plastic surgery department managed complex fractures involving joint surface involvement and ligamentous injuries with arthroscopic assistance.

This study compares the cost structure and cost-effectiveness of fluoroscopy-assisted ORIF and arthroscopy-assisted ORIF, taking into account DRF severity (AO classification) and treatment indications from the literature, to assist surgeons and healthcare providers in making more informed decisions regarding DRF treatment options.

## 2. Materials and Methods

This monocentric retrospective study was conducted between October 2021 and March 2022 at Ludwig-Maximilians-University (LMU) Hospital, Munich, Germany. Inclusion criteria were a medical indication for surgical treatment of a DRF using volar plate osteosynthesis and the performance of this procedure, as well as a patient age of over 18 years. General exclusion criteria were a DRF without an indication for surgical treatment, a patient’s refusal of surgical treatment, treatment of the DRF using any method other than volar plate osteosynthesis, and a patient’s age under 18 years. Ethical approval for this study was granted by the ethics committee of the LMU (No. 22-0674, Date: 26 August 2022, with Addendum, Date: 15 August 2023).

### 2.1. Conditions of the Study Implementation

This study was conducted at a subsidiary site of the university clinic, which is certified as a supraregional hand trauma center. When indicated for surgery, distal radius fractures were treated by the Department of Hand Surgery, Plastic Surgery, and Aesthetic Surgery, as well as the Department of Orthopaedics and Trauma Surgery. According to lost internal clinic agreements, complex intra-articular fractures with signs of accompanying ligamentous injuries are mainly treated by plastic surgeons using arthroscopically assisted methods. Extra-articular or minimally displaced fractures are treated by orthopedic surgeons. For polytraumatized patients also presenting with a DRF, treatment is usually provided by the orthopedic/trauma department. Both the Department of Hand Surgery, Plastic Surgery, and Aesthetic Surgery, as well as the Department of Orthopaedics and Trauma Surgery, treated intra-articular fractures, whereas in our hospital, only the Plastic Surgery Department performed arthroscopically assisted fracture management. Patients included in this study were referred either by outpatient physicians or presented via the in-house emergency departments and trauma bay, respectively.

### 2.2. Indication and Treatment

The fractures were classified according to the AO classification based on preoperative X-rays and CT scans, as assessed by trained hand surgeons [23]. The decision to perform simultaneous wrist arthroscopy was based on fracture classification, displacement, and intraoperative reduction results or preoperatively suspected concomitant ligament or cartilage injury [13,24,25]. All osteosynthetic treatments of the DRF were performed via ORIF using volar plate osteosynthesis (Medartis AG, Basel, Switzerland) [3,6,7,26]. The procedures performed during osteosynthesis and arthroscopy were documented from the surgical reports.

### 2.3. Study Design

**Figure 1:**
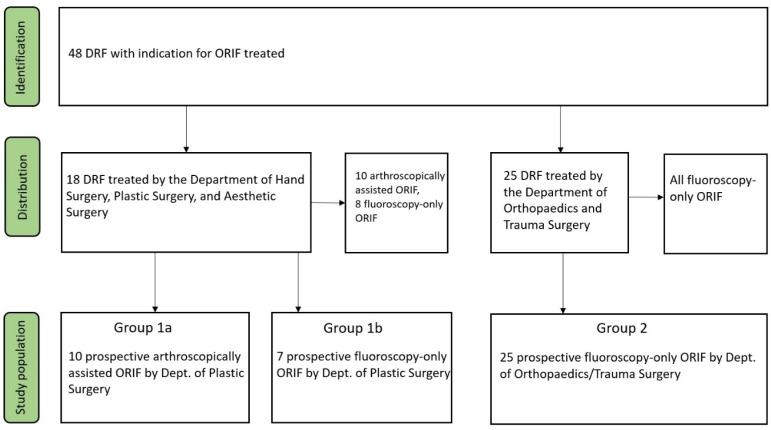
Study flow chart.

The study population was divided according to the treating department (Group 1 and Group 2, respectively). Additionally, Group 1 was further subdivided into Group 1a and Group 1b based on the type of treatment, with arthroscopic assistance and purely fluoroscopic ORIF, respectively (Figure 1).

### 2.4. Analysis

The economic analysis was conducted based on two main aspects: first, the cost breakdown of standard surgical trays, single-use items, and other necessary materials used for the procedures [18,27]. Second, the cost of the average surgical minute was calculated, distinguished by the type of treatment and the department performing the procedure. Only the running costs of the surgical interventions were considered in this study; investment costs for equipment and infrastructure were not taken into account. The data were provided and approved by the in-house Department of Controlling.

In Germany, reimbursement for inpatient (surgical) treatment of patients is based on DRGs. These are determined using the diagnosis (according to ICD-10), the treatment performed (e.g., OPS code), and, if applicable, additional factors. The reimbursement for distal radius fractures was exemplarily determined using the Webgrouper tool by the DRG Research Group (www.drg-research-group.de (accessed on 15 April 2025), DRG Research Group, Senden, Germany) (See Table 1).

The data are presented as means with standard deviation or as absolute and relative values unless otherwise specified. Normal distribution was assessed using the Shapiro–Wilk test. All calculations were performed using SPSS Statistics 28 (IBM, Armonk, NY, USA). Results were considered statistically significant at a probability level of ≤0.05. Graphs were created using Microsoft Excel MSO 2019, Version 1808 (Microsoft, Redmond, WA, USA).

## 3. Results

Between October 2021 and March 2022, a total of 43 DRFs were treated. Of these, 17 procedures were performed by the Department of Hand Surgery, Plastic and Aesthetic Surgery (Group 1), of which 10 were arthroscopically assisted (Group 1a) and 7 were performed via fluoroscopy only (Group 1b). Additionally, 25 fracture treatments were performed by the Department of Orthopaedics and Trauma Surgery (Group 2).

The average age in Group 1 was 49.6 years (SD = 19.4), with a 64% majority of female patients. In Group 1a, the average age was 53.9 (SD = 16.3) with 60% female patients and 40% male patients, and in Group 1b, the average age was 43.6 (SD = 23.1) with 71.4% female patients. The average age in Group 2 was 54.2 years (SD = 21.0), with 36% male patients and 64% female patients. There was no significant difference in age and gender distribution within the groups and subgroups (*p* > 0.05).

**Figure 2:**
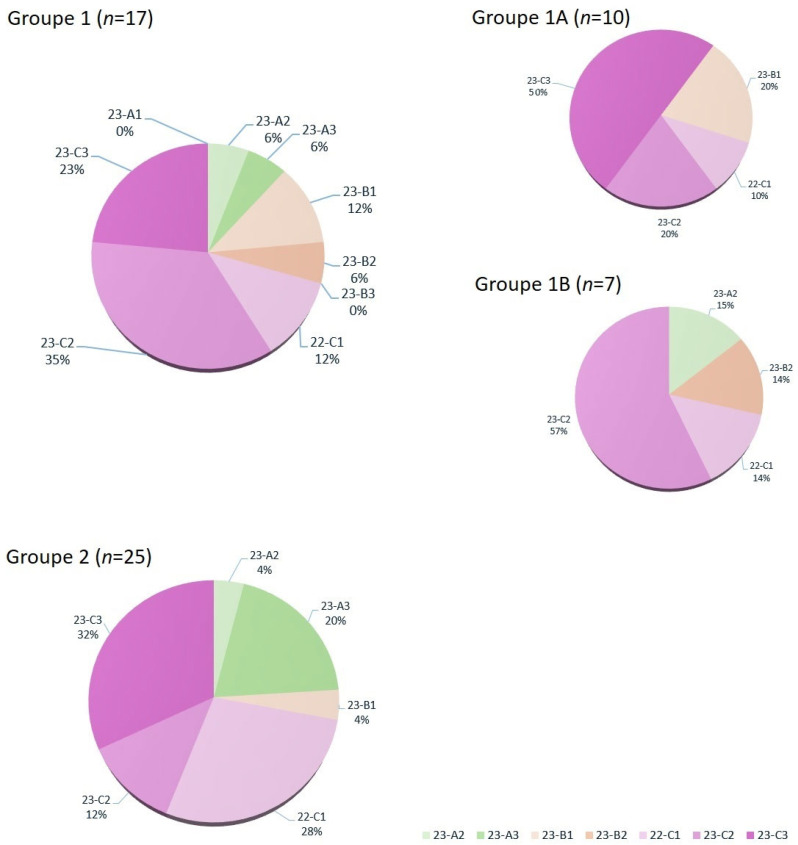
Distribution of the number of fractures by severity according to the AO classification across the different groups: Group 1—Plastic Surgery (all), Group 1A—Plastic Surgery (arthroscopically assisted), Group 1B—Plastic Surgery (fluoroscopic only), and Group 2—Orthopaedics and Trauma Surgery (all).

The Department of Plastic Surgery treated a total of seven DRFs via volar plate osteosynthesis using fluoroscopic reduction only (Group 1b). In 10 cases, the intra-articular involvement or fragment displacement was so severe that arthroscopically assisted treatment was necessary (Group 1a). In this Group, diagnostic arthroscopy is used to verify the step-free restoration of the distal radial joint surface. Additionally, debridement using a shaver was performed in seven cases (38.8%), the wrist was flushed, and loose joint bodies were removed. In two cases (11.1%), improved joint surface alignment was achieved through arthroscopic fragment fixation. In one case, a TFCC lesion (Palmer 1B/Atzei 1) was diagnosed, and in another case, the diagnosis of a fresh SL ligament injury led to an open SL ligament repair. Of the 25 DRFs treated in Group 2, 19 were intra-articular (76%) and 6 were extra-articular fractures. All fractures were treated with open reduction and internal fixation using a volar plate. No arthroscopically assisted procedures were performed by orthopedic hand surgeons. The fracture severity according to the AO classification across the different groups is shown in Figure 2.

## 4. Procedure Costs

Table 1 and Group 2: In Group 1, the cost for standard surgical draping, such as the treatment of a distal radius fracture, is EUR 216.61, whereas in Group 2, it is only EUR 176.52. The reason is mainly the additional use of a handset for EUR 27.67, an extra small adhesive drape for EUR 0.59, and an adhesive cover drape for EUR 1.19. For the fluids used, there is also a price difference of EUR 1.59 in favor of Group 2. The costs arising from the use of reusable sterile items are higher in Group 1, with the surgical trays being as follows: the “Hand-tray” (EUR 38.50), the “Medartis-Radius-tray” (EUR 32.50), and the Drill-Tray “Colibri” (EUR 38.50). Most of the time, Group 1 requires the standby of wrist arthroscopy. If indicated in the surgical preoperative plan, an additional “Wrist Arthroscopy Set” with a Shaver and a 2.4× optic is prepared, which incurs additional costs of EUR 17.50 and EUR 3.15, respectively, regardless of its actual intraoperative use. Group 1 uses the Orthoscan (Ziehm Imaging GmbH, Nuremberg, Germany) for intraoperative fluoroscopy, whose sterile coverage is EUR 3.22 more expensive than the coverage of the Vista C-Arm (Ziehm Imaging GmbH, Nuremberg, Germany) used by Group 2. Additionally, Group 1 routinely uses more expensive suture material, costing EUR 11.36, compared to Group 2’s suture material costing EUR 9.20. Dressing materials contribute EUR 6.80 to the total cost in Group 1 and EUR 3.26 in Group 2. Both groups use Medartis volar plates and cortical and angular-stable screws of this brand. The total cost for draping materials and other single-use items amounts to EUR 216.61 and EUR 176.52 for the Department of Plastic Surgery and the Department of Orthopaedics and Trauma Surgery, respectively (see Table 2).

Both departments use osteosynthesis material from Medartis (Basel, Switzerland) by default. For material costs, the prices of the plates (Classic styloid-oriented volar plate and FPL plates) [26] and the screws used were summed up. In total, the cost of the material used for osteosynthesis amounts to EUR 450.00 (SD: 43.46) for Group 1A and EUR 446.82 (SD: 24.59) for Group 1B, compared to EUR 453.16 (SD: 34.04) in Group 2. The cost difference for the osteosynthesis material used was not significant.

Overall, the material costs in the Department of Plastic Surgery amounted to EUR 665.30, while in the Department of Orthopaedics and Trauma Surgery, they amount to EUR 629.68. With a difference of EUR 35.06, the difference between the two departments was not significant (*p* = 0.7268). Even when comparing Group 1B, including the cost of arthroscopy equipment, the pure material costs of arthroscopy-supported ORIF performed by plastic surgeons (EUR 663.43, SD: 24.58) are not significantly higher than the costs for fluoroscopically guided ORIF performed by orthopedic surgeons (*p* = 0.0209).

Costs per surgical minute were determined by adding the overhead costs, operating costs of the OR, and personnel costs of all 14 operating areas at the University Hospital Munich. For the year 2021, this resulted in an average cost of EUR 41.76 per surgical minute (in-house Department of Controlling). Surgical procedures in Group 1 lasted on average 98.5 (SD = 37.3) minutes (incision-to-closure time). In the subgroup analysis of 1a and 1b, the average operation duration was 80.1 (SD = 27.5) minutes for fluoroscopy-only fracture treatment (Groupe 1b) and 111.5 (SD = 39.0) minutes for arthroscopically assisted treatment of the distal radius fracture (Groupe 1a). Among the procedures performed by plastic surgeons, there was a significantly longer duration when arthroscopic assistance for the osteosynthesis was necessary (*p* = 0.0420). In Group 2, the procedures took an average of 65.0 (SD = 34.5) minutes. Therefore, the duration for fluoroscopy-only ORIF of the distal radius fracture performed by orthopedic surgeons is significantly shorter than the average procedure time in Group 1 overall (*p* = 0.0048). Comparing the procedure times of subgroups 1a and 1b with Group 2, there is a significantly shorter procedure time of 50.5 min for orthopedic treatment (Group 2) compared to arthroscopically assisted treatment (Group 1a) (*p* = 0.0015). However, there is no significant difference when comparing the classic fluoroscopy-only treatment between the specialties (Group 2 vs. Group 1b) (*p* = 0.2994).

**Figure 3:**
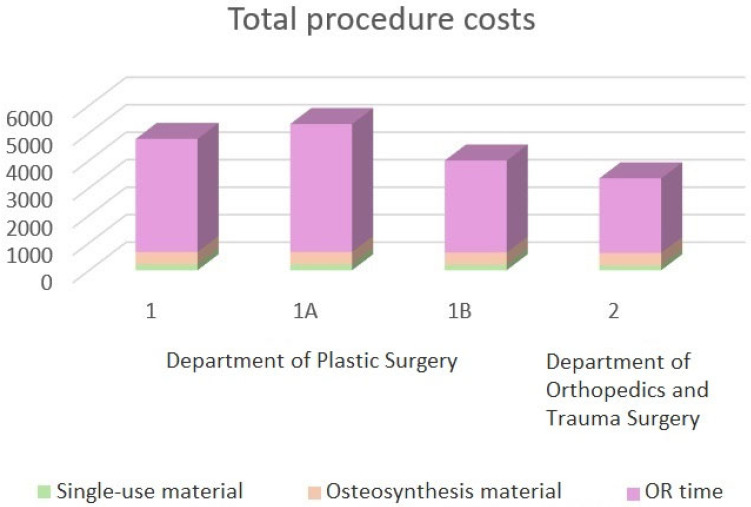
Total costs of DRF ORIF (running costs only). Single-use material, osteosynthesis material, and OR time are summed up for each analyzed group.

With regards to the costs, the total costs in Group 1, consisting of standard costs, operating minute costs, and material costs, amount to an average of EUR 4906.58. In the subgroup analysis, the average costs for arthroscopically assisted treatment the costs amounted to EUR 5448.24 (Group 1a), and the conventional open reduction and internal fixation (Group 1b) were EUR 4132.80. The costs in Group 2 amount to an average of EUR 3344.08 (Figure 3).

## 5. Reimbursement

In Germany, reimbursement for inpatient services is based on DRG (Diagnosis-Related Groups). The base case value in 2022 was EUR 4400.00. The possible diagnoses for distal radius fractures (ICD-10) and surgical treatment options (OP codes) are listed in Table 1. To determine the minimum reimbursement for the treatment of a DRF according to DRG, the reimbursement was exemplarily calculated using the Webgrouper tool by the DRG Research Group. All ICD-10 codes (S52.50, S52.51, S52.52, and S52.59) used for the cases included in this study were applied and combined with the corresponding OPS codes (5-793.36 and 5-794.26), including the code for arthroscopically assisted fracture treatment (5-810.68). Possible additional procedures, independent of the distal radius fracture, were not taken into account.

The result for each combination was a reimbursement under I21Z. The DRG showed an effective DRG weighting of 0.866, which in 2022 corresponded to EUR 3810.40. Neither the indication-determining severity of the fracture according to the AO classification, nor a possible malalignment of fracture fragments, nor the additional effort of arthroscopy-assisted osteosynthesis, was reflected in the reimbursement.

**Figure 4:**
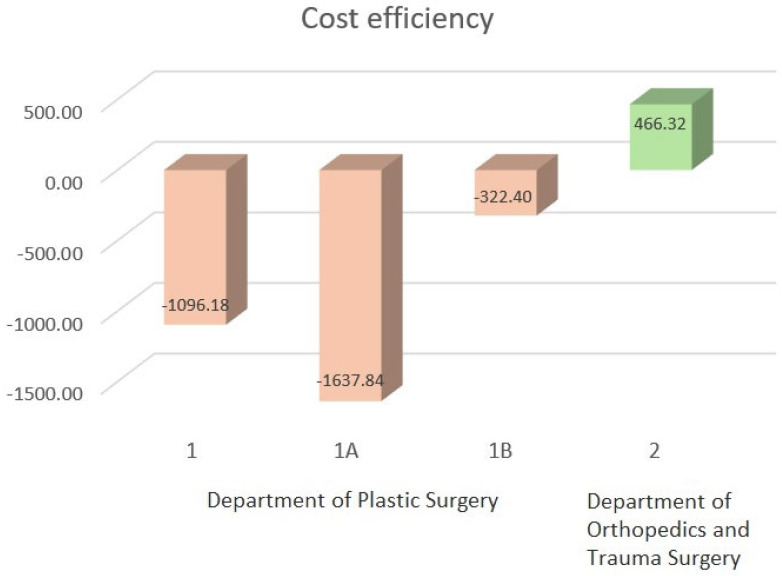
Cost coverage of operative inpatient treatment of DRF according to DRG in the various analyzed groups (reimbursement minus total running procedure costs). The red bars represent deficient procedures, while the green bar indicates cost-effective interventions. Accommodation and patient catering are not included.

When the costs of treating a DRF with volar plate osteosynthesis are compared to the reimbursement, it becomes evident that the reimbursement for complex fractures treated by the Department of Plastic Surgery is not cost-covering. In particular, arthroscopically assisted treatment is significantly more expensive than the granted reimbursement. Only the conventionally fluoroscopy-guided osteosyntheses performed by the Department of Orthopaedics and Trauma Surgery are barely cost-covering due to the shorter operative time (Figure 4). For full reimbursement by the health insurance providers, patients must remain in inpatient treatment for at least two calendar days. However, the accommodation and meal costs already covered by the DRG (§ 17d KHG) have not yet been considered in this calculation.

## 6. Discussion

This study aimed to compare the cost structure and cost-effectiveness of fluoroscopy-assisted and arthroscopy-assisted open reduction and internal fixation (ORIF) for treating distal radius fractures (DRFs). Previously conducted studies primarily compare postoperative outcomes and often describe the use of arthroscopy as costly, without specifying the actual amount or the reason for these costs [28,29,30]. To our knowledge, a direct comparison of the additional costs of arthroscopically assisted versus purely fluoroscopy-guided ORIF for distal radius fractures (DRFs) has not yet been conducted.

The cost analysis of DRF treatment using volar plate osteosynthesis revealed only slight variations in material costs between the Department of Hand Surgery, Plastic Surgery, and Aesthetic Surgery, and the Department of Orthopaedics and Trauma Surgery, respectively. The differences were primarily due to variations in surgical draping standards and the use of single-use materials. Even though the differences were minimal, the Department of Orthopaedics and Trauma Surgery utilizes slightly more cost-effective materials. Importantly, additional costs associated with arthroscopy were not significant when compared to fluoroscopy-only treatments.

The largest proportion of total costs was attributed to OR usage. Operational costs were calculated per surgical minute and multiplied by the average operation duration for each group. The analysis showed a significantly shorter operation time in the Department of Orthopaedics and Trauma Surgery compared to the Department of Plastic Surgery. In particular, arthroscopically assisted DRF treatments had a significantly longer operation time, resulting in higher costs.

Due to the varying reimbursement models in different national healthcare systems, no universally valid conclusion can be drawn regarding the cost-effectiveness of arthroscopically assisted treatment of intra-articular DRFs [31,32]. The cost-effectiveness assessment, therefore, refers specifically to the conditions within the German healthcare system. Since treatment of DRFs via ORIF is not part of the German Mandatory Outpatient Surgeries Catalogue (Katalog ambulant durchführbarer Operationen), thus is usually performed as an inpatient service [20]. The inpatient treatment of DRFs is reimbursed according to the DRG system. The analysis revealed that reimbursement is both independent of fracture severity (AO classification or displacement) as well as the potential need for arthroscopically assisted fracture treatment. In all cases, reimbursement was classified under DRG I21Z with an effective DRG factor of 0.866, which corresponded to EUR 3810.40 in 2022 (this study’s endpoint). This reimbursement stands in contrast to the costs of an average of EUR 5448.24 for arthroscopically assisted treatment, and EUR 4132.80 or EUR 3344.08 for fluoroscopy-only ORIF. The reimbursement is therefore barely or not cost-covering for the running costs of the actual treatment. Further costs, such as inpatient catering and accommodation, were not considered.

The gold standard for surgical treatment of intra-articular DRFs is ORIF with volar plate osteosynthesis, which achieves excellent outcomes [2,3,6,7]. In particular, for intra-articular fractures with displaced or difficult-to-reduce fragments, as well as suspected ligamentous or chondral concomitant injuries, accompanying wrist arthroscopy is indicated and can lead to improved postoperative outcomes and allow simultaneous treatment of potential concomitant injuries [5,9,10].

The indication for arthroscopic assistance was based on the preoperative clinical appearance and imaging. For this study, preoperative X-ray and CT images were classified according to the AO classification by experienced hand surgeons. Since the AO classification does not necessarily reflect an increasing severity of the fracture, evaluating the average fracture severity within the analyzed groups proved challenging. It was therefore decided to present the number of fractures graphically and compare them in the discussion.

Both the Department of Hand Surgery, Plastic Surgery, and Aesthetic Surgery, as well as the Department of Orthopaedics and Trauma Surgery, treated intra-articular and non-intra-articular fractures. The relatively high proportion of intra-articular fractures can most likely be attributed to the fact that simpler fractures are often initially reduced and then treated conservatively. Intra-articular fractures more frequently require surgical reduction to achieve a good postoperative outcome. In a subgroup analysis of Group 1 (Department of Plastic Surgery), a significant difference in fracture severity was observed. In Group 1a (arthroscopically assisted), only intra-articular fractures (B1-C3) were found, whereas in Group 1b (treated with fluoroscopy only), both intra-articular fractures and fractures of type A2 and A3 were present. Group 1b closely resembled the Orthopedic/Trauma-Surgical Group 2 in terms of fracture type distribution. A higher frequency of more severe (higher-grade fractures according to the AO classification) was also observed in group 1a. In addition to the more frequent indication for arthroscopy resulting from this, the higher complexity of the fractures likely also contributed to the prolonged operation time and, thus, the significantly higher costs in this group.

Limitations of the study include the limited study population with a small number of cases in each group. Additionally, the monocentric approach may present a one-sided view of the cost structures. Furthermore, there is difficulty in objectively grading the severity and, thus, the potential surgical time required for the individual fractures, making them challenging to compare. Larger trials should be conducted.

Due to the complexity of the fractures and possible concomitant injuries, the treatment of intra-articular radius fractures should, from a medical perspective, be centralized at specialized hand trauma centers. From an economic perspective, this also seems sensible, as the high caseload and increased routine can help reduce operation times, particularly in arthroscopically assisted treatment. Although the results of this study question the cost-effectiveness of arthroscopically assisted treatment for intra-articular DRFs, the authors, from a hand surgery perspective, are convinced of the better outcomes with this technique and continue to use it for the benefit of the patients.

## 7. Conclusions

Intra-articular DRFs with severely displaced fragments or concomitant injuries benefit from arthroscopically assisted fracture treatment. Arthroscopically assisted and fluoroscopy-only treatments do not differ significantly in material costs; however, the procedures performed with arthroscopic assistance take significantly longer. This results in the largest portion of the costs for performing DRF treatment. Unfortunately, this increased time expenditure is not reflected in the reimbursement through the DRG system. Overall, all variants of fracture treatment are either not or only barely cost-covering. When additional factors for the compulsory inpatient stay are included, like catering and accommodation, the reimbursement is insufficient to perform DRF treatment in a cost-effective manner. As outpatient treatment continues to increase, a significantly adjusted reimbursement is necessary to perform these procedures economically.

## Figures and Tables

**Table 1:** Potential reimbursement by DRG for different types of distal radius fractures included in this study and their respective treatments (Example: calculation using the Webgrouper tool by the DRG Research Group, Senden, Germany).

ICD-10	OPS	DRG
S52.50 Distal radius fracture, unspecified	5-793.36 (open reduction of a simple fracture in the joint area: distal radius) or 5-794.26 (open reduction of a multi-fragment fracture/multi-fragment joint fracture: distal radius)	I21Z (0.866 effective DRG valuation ratio = EUR 3810.40)
S52.51 Distal radius fracture, extension fracture	5-793.36 or 5-794.26	I21Z (0.886 effective DRG valuation ratio = EUR 3810.40)
S52.52 Distal radius fracture, flexion fracture	5-793.36 or 5-794.26	I21Z (0.866 effective DRG valuation ratio = EUR 3810.40)
S52.59 Distal radius fracture, other and multiple parts Incl.: Intra-articular fracture	5-793.36 or 5-794.26	I21Z (0.866 effective DRG valuation ratio = EUR 3810.40)
S52.59 Distale Radiusfraktur, other and multiple parts Incl.: Intra-articular fracture	5-793.36 or 5-794.26 and 5-810.68 (Arthroscopically assisted treatment of a fracture: Radiocarpal joint)	I21Z (0.866 effective DRG valuation ratio = EUR 3810.40)

**Table 2:** Detailed list of draping materials and other single-use items according to the department standards in Plastic Surgery and Orthopaedics and Trauma Surgery, respectively. Prices are listed in Euro.

Department of Plastic Surgery	Price (Euro)	Department of Orthopaedics and Trauma Surgery	Price (Euro)
Absorbent cloth	0.18	Absorbent cloth	0.18
Surgical drapes		Surgical drapes	
Hand set	27.67	Hand set	27.67
Small drape	0.59		
Drape with adhesive tape	1.19		
Surgical trays		Surgical trays	
Hand tray	38.50	Hand tray	38.50
Medartis Radius Tray	32.50	Medartis Radius Tray	32.50
Colibri Drill Tray	38.50	Colibri Tray	38.50
Lamp handle	1.15	Lamp handle	1.15
Arthroscopy set and Shaver	17.50		
2.4 optic	3.15		
Surgical instruments		Surgical instruments	
gloves	0.93	gloves	0.93
15 blade	1.96	15 blade	0.98
Syringe 10 mL	0.04	10 blade	0.29
Pen	2.37	Pen	2.37
Vacuum	2.36	Vacuum	2.36
Vacuum tip	0.52	Vacuum tip	0.52
Stool cover	0.94	Stool cover	0.94
Pressure cuff	11.50	Pressure cuff	11.50
Orthoscan cover	10.71	X-ray cover	7.49
Surgical threads		Surgical threads	
Vicryl UCL 2-0	3.91	Polysorb 2-0	1.74
Vicryl 4-0	5.87	Surgipro 2-0	1.14
Prolene 3-0	1.68		
Wound dressing		Wound dressing	
Elastic bandage 6 cm	3.18	Elastic bandage 6 cm	3.18
Bandaid	0.08	Bandaid	0.08
Wound gaze	0.26		
Cast	3.28		
Liquids		Liquids	
Ringer’s solution	1.04	Kodan colorred	4.50
Kodan colourless	5.05		
Total	216.61	Total	176.52

## Data Availability

The original data presented in this study are openly available in PubMed at DOI.
